# Acculturation does not necessarily lead to increased physical activity during leisure time: a cross-sectional study among Turkish young people in the Netherlands

**DOI:** 10.1186/1471-2458-7-230

**Published:** 2007-09-03

**Authors:** Karen Hosper, Niek S Klazinga, Karien Stronks

**Affiliations:** 1 Department of Social Medicine, Academic Medical Centre – University of Amsterdam, P.O. Box 22700, 1100 DD Amsterdam, the Netherlands

## Abstract

**Background:**

Non-Western migrant populations living in Western countries are more likely to be physically inactive during leisure time than host populations. It is argued that this difference will disappear as they acculturate to the culture of the host country. We explored whether this is also true for migrants who experience contextual barriers such as having children, living in a less attractive neighbourhood, or having occupational physical activity.

**Methods:**

Cross-sectional data were obtained from the LASER-study (2003–2004) on health related behaviours in first and second generation Turkish young people living in the Netherlands. For this study we included 485 Turkish participants aged 15–30 years, who participated in a structured interview during a home visit. Acculturation was indicated by level of 'cultural orientation towards the Dutch culture' and 'social contacts with ethnic Dutch' with persons being low oriented towards the Dutch culture and having few social contacts with ethnic Dutch as reference group. The measured barriers were 'having children', 'occupational physical activity' and 'living in a less attractive neighbourhood'. Logistic regression analyses were used to assess the associations between acculturation and physical activity during leisure time, stratified by these contextual barriers.

**Results:**

Greater cultural and social integration was associated with increased physical activity during leisure time. Odds ratio's were 1.85 (CI: 1.19–2.85) for 'cultural orientation' and 1.77 (CI: 1.15–2.71) for 'social contacts with ethnic Dutch'. However, these associations were not present or less strong among people who had children, or who were living in a less attractive neighbourhood or who engaged in occupational physical activity.

**Conclusion:**

Physical activity during leisure time increased with greater acculturation, however, this relationship was found only among participants without children, living in a attractive neighbourhood and having no occupational activity. Interventions aimed at migrant populations should not only focus on the least integrated. Instead, effectiveness might be enhanced when interventions are sensitive to the contextual barriers that might inhibit physical activity behaviours during leisure time.

## Background

Physical inactivity is currently acknowledged to be a serious public health burden in the industrialized world [1,2]. A large body of evidence shows that regular physical activity reduces the risk of death from several conditions including coronary heart disease, hypertension, obesity and diabetes type II [3-7]. Despite the known beneficial health effects of physical activity, two- thirds of the population living in Europe does not achieve the minimum recommended amount of physical activity [8]. While the level of inactivity among the general population is high, non-Western migrants living in Western countries have an even greater risk of being physically inactive [9-12]. This applies, amongst others, to the Turkish migrants in Western European countries, such as the Netherlands and Sweden [13-16].

Previous studies have indicated that the level of physical activity among migrant populations converges towards the level in the host population with greater language fluency, increasing numbers of stay in the host country or with increasing generational status [17-20]. These factors are considered to be indicators of the level of 'integration' into the host society, which is often referred to as the process of acculturation [21]. One of the earliest definitions of acculturation is as follows: "Culture change that is initiated by the conjunction of two or more autonomous cultural systems. Its dynamics can be seen as the selective adaptation of value systems, the process of integration and differentiation (..)" [21].

Parallel to the studies on acculturation, attention has increasingly focused on barriers for physical activity resulting from the social and environmental context, as is often embedded in ecological models [22-24]. It has, for example, been shown that people living in neighbourhoods with few sidewalks, a high volume of traffic, no aesthetic attributes or high crime, i.e. a less attractive neighbourhood, are less likely to engage in physical activity than those who live in more attractive and more exercise-supportive areas [24-31]. Moreover, having children can function as a time-barrier for physical activity. Child care, often in combination with household activities, decrease the opportunities to be physically active, in particular during leisure time [32-34]. Furthermore, engaging in physical activity at work has also been found to have a negative influence on leisure time activity [35].

However, the studies on the association between acculturation and physical activity have largely neglected the effect of contextual barriers. This might be seen as an omission in these studies, as most contextual barriers are in general even more prevalent among migrant than among host populations, due in part to the lower socioeconomic status of most migrant populations [11,36].

**Figure 1:**
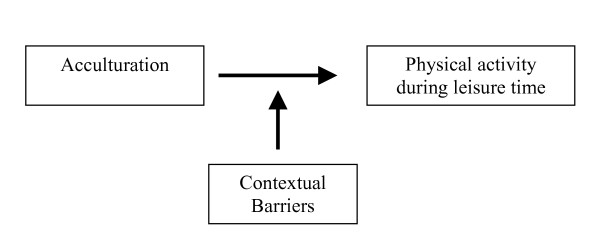
An illustration of the studied associations.

Therefore, the objective of our study was to gain insight into how the association between physical activity during leisure time and acculturation, measured by cultural and social integration, might be modified by the following contextual barriers: having children, occupational physical activity or living in a less attractive environment. Figure 1 illustrates the associations we studied. We analysed this among the Turkish population in the Netherlands, one of the largest ethnic minorities in several other Western European countries as well [37,38].

## Methods

Cross-sectional data were obtained from the LASER study (Lifestyle in Amsterdam: Study among Ethnic gRoups) on prevalence and determinants of health-related behaviour among two non-Western migrant populations in the Netherlands, including the Turkish migrants. Data collection took place from April 2003 until December 2004. This study has been approved by the Medical Ethical Commission of the Academic Medical Centre in Amsterdam, the Netherlands.

A random sample was drawn from the Amsterdam population registry which included people born in Turkey or people who were born in the Netherlands, but have one of both parents being born in Turkey. This implies that the term 'migrants' as used in this study, includes individuals of the first as well as of the second generation. Participants received a letter of invitation, including a translated version in Turkish. Interviewers of Turkish origin and of the same sex as the participant, visited the participants' homes and requested their cooperation for an interview. A written informed consent was obtained from each participant in the study.

A structured questionnaire was used that included questions about health-related behaviour and determinants, among which socio-economic position, migration history and acculturation. Prior to the study, the questionnaire had been forward and back-translated by professional Turkish translators. Translations were discussed with the researcher and the translators to ensure that the meaning of the questions did not change.

The total sample consisted of 1556 persons with a Turkish origin, aged 10–30 years. Approximately 13% could not be traced because of incorrect address information. Of the 1354 respondents that could be traced, 768 participated in the study (57 %). Most cases of 'non response' were refusals to participate (32 %) or the persons could not be reached after three attempts (12 %).

For the current study we only included participants of 15–30 years of age, resulting in 236 men and 249 women. We used 15 years as our lower age cut-off point because we expected acculturation to have less effect on physical activity among children below this age [39]. In addition, the greatest decline in physical activity is generally found in adolescence between 13–16 years of age [40,41].

### Physical activity during leisure time

Physical activity during leisure time was assessed using one component of the Short QUestionnaire to ASsess Health enhancing physical activity (SQUASH) which includes questions about sports and other leisure time activities like walking, cycling, gardening and doing odd jobs during leisure time [42]. This instrument is comparable with the International Physical Activity Questionnaire (IPAQ) [43]. Dancing was added to the questionnaire since it is a common activity among Turkish girls and women. Total minutes of activity were calculated by multiplying frequency (days/week) by duration (min/day). Activity scores for separate questions were calculated by multiplying total minutes of activity by the intensity score. All activities were coded according to the Compendium of Physical Activities of Ainsworth [44]. The intensity score was expressed in MET's (i.e. metabolic equivalent or number of times resting metabolic rate). Subjects were classified as being active *during leisure time *when they undertook at least 30 minutes (or 60 minutes for people under the age of 18) of moderate activity per session at least one day a week. The main reason for this definition is the fact that more than half of the participants were not participating in any leisure time physical activity. Therefore, we considered the distinction between any activity versus none relevant for this population.

For people aged younger than 18 years, the cut-off point for moderate activity was 5 MET or higher and 4 MET or higher for people aged 18 and older [45].

### Indicators of acculturation

The indicators of acculturation were based on Berry's approach whereby acculturation is considered in terms of orientation towards the majority culture versus culture of origin and social contacts with the host population versus contacts with people from culture of origin [46]. This resulted in the following two components:

*'Cultural orientation' *was measured by 10 items about language use with family members and friends, use of media, difficulties with reading the Dutch language, shopping preferences and emancipation as an example of Western norms and values [47,48]. The cultural orientation scale was constructed using principal component and reliability analysis (alpha = 0.64).

*'Social contacts with ethnic Dutch' *were measured by three questions about contacts with ethnic Dutch people during leisure time (alpha = 0.84).

The scores on the items in each scale were summed and a mean substitution was made for cases where one item was missing. To distinguish between the lower and the higher acculturated persons, the upper third was categorized as being high acculturated and the remaining two third as being low acculturated. Using this categorization we had large enough numbers of participants to perform stratified analyses.

### Contextual barriers

*Physical environment of the neighbourhood *was assessed by a 19-item list containing neighbourhood characteristics on which participants could agree or disagree on a 4-point scale [49]. The variables included availability of sidewalks, availability of cycle lanes, neighbourhood aesthetics, perceived safety from crime and perceived safety from traffic. People who scored negatively on 8 or more items were categorized as living in a less attractive environment.

*Occupational physical activity *was measured by one question about how many hours a week people were engaging in physical activity at work such as walking a lot or carrying heavy loads. Participants were categorized as being active at work when they participated for at least 30 minutes a day (5 days a week) in occupational activities with moderate intensity (≥4.0 MET) [44].

*Children under 16*: Participants were asked if they had children under the age of 16 living with them.

*Exercise inhibiting or supportive context*: we added one overall measure of contextual barriers by dividing the participants into people who experienced at least one of the mentioned barriers (exercise inhibiting context) versus people who experienced none of the mentioned barriers (exercise supportive context).

### Analysis

Logistic regression analysis (SPSS 12.0.1 for windows) was used to assess the association between both indicators of acculturation and leisure time physical activity. All analyses were adjusted for age, sex, education and marital status. To assess whether these associations differed by the presence of contextual barriers (having children, occupational physical activity, less attractive neighbourhood), we conducted stratified analyses [50].

## Results

### Description of the sample

Of the total study population 44% was born in Turkey (first generation) and 56% in the Netherlands with at least one parent born in Turkey (second generation). Regarding the contextual barriers, approximately one-third of the participants perceived their neighbourhood environment as less attractive or had children under the age of 16. The barrier of occupational physical activity was found among only 16% of the participants (Table 1). Approximately 45% of all participants did not participate in any physical activity during their leisure time.

**Table 1:** Characteristics of the study population with percentage of the participants who are active during leisure time for each characteristic

	**Total number of participants** ** N = 485** **n (%)**	**Physically *in*-active participants** ** n (%)**	**Physically active participants ** **n (%)**
**Sex**			
Men	236 (48.7)	87 (40.3)	149 (55.4)*
Women	249 (51.3)	129 (59.7)	120 (44.6)
**Age**			
15–19 years	221 (45.6)	99 (45.8)	122 (45.4)
20–24 years	113 (23.3)	44 (20.4)	69 (25.7)
25–30 years	151 (31.1)	73 (33.8)	78 (29.0)
Mean age (sd)	21.6 (5.0)	21.8 (5.2)	21.5 (4.8)
**Country of birth**			
Born in Turkey	212 (43.7)	105 (48.6)	107 (39.8)*
Born in the Netherlands	273 (56.3)	111 (51.4)	162 (60.2)
**Marital status**			
Married or cohabiting	167 (34.4)	89 (41.2)	78 (29.0)*
Not married/not cohabiting	318 (65.6)	127 (58.8)	191 (71.0)
**Educational level**			
Low	226 (46.6)	107 (49.5)	119 (44.2)
Moderate to high	259 (53.4)	109 (50.5)	150 (55.8)
**Acculturation indicators^a^**			
*Cultural orientation*			
Low	304 (65.8)	151 (72.2)	153 (60.5)*
High	158 (34.2)	58 (27.8)	100 (39.5)
*Social contacts with ethnic Dutch*			
Few	346 (72.2)	169 (79.0)	177 (66.8)*
Many	133 (27.8)	45 (21.0)	88 (33.2)
**Contextual barriers**			
*Neighbourhood environment^a^*			
Attractive neighbourhood	299 (64.7)	120 (58.5)	179 (69.6)*
Less attractive neighbourhood	163 (35.3)	85 (41.5)	78 (30.4)
*Children under 16*			
Having no children	322 (66.4)	134 (62.0)	188 (69.9)*
One or more children living at home	163 (33.6)	82 (38.0)	81 (30.1)
*Occupational physical activity^a^*			
No activity at work	345 (83.9)	204 (94.4)	241 (89.6)*
Activity at work	66 (16.1)	12 (5.6)	28 (10.4)
**Overall measure of contextual barriers**^b^			
Exercise-supportive (no barriers)	197 (40.6)	73 (34.9)	116 (44.3)*
Exercise-inhibiting (≥1 barrier)	288 (59.4)	136 (65.1)	146 (55.7)
**Physical activity during leisure time**			
Physically active (≥ once a week)	269 (55.5)		
Physically *in*active	216 (44.5)		

^a ^Numbers do not add up to total number of sample due to missing data.

^b ^When none of the three barriers (children, less attractive neighbourhood, occupational physical activity) were present, people were categorized as living in an exercise-supportive context. When one or more barriers were present, people were categorized as living in an exercise-inhibiting context.

* P-value < .05

Table 1 also shows the characteristics according to the participants' level of activity. As expected, the participants with a greater orientation towards the Dutch culture or having more contacts with ethnic Dutch were more often physically active during leisure time than the participants who were less oriented towards the Dutch culture. Participants living in an attractive neighbourhood and the participants without children were more often physically active during leisure time than the participants who did have these barriers. For the overall measure of contextual barriers we also found that the participants with one or more of the mentioned barriers were less often physically active during leisure time than the participants with none of these barriers. For occupational physical activity we found no significant associations.

### Associations of the acculturation indicators with physical activity during leisure time

Table 2 shows the associations between acculturation and physical activity during leisure time expressed in Odds Ratio's (OR) with 95% Confidence Intervals (CI). These associations are adjusted for age, sex, education and marital status. Both indicators of acculturation were positively associated with physical activity during leisure time. The odds for 'cultural orientation' was 1.85 (CI: 1.19–2.85) and 1.77 (CI: 1.15–2.71) for social contacts (Table 2).

**Table 2:** Associations^a ^of the indicators of acculturation with physical activity during leisure time

	**Physical activity during leisure time** ** N = 485 ** **OR (95% CI)**
**Acculturation indicators**	
*Cultural orientation towards Dutch culture*	
Low cultural orientation	1.00
High cultural orientation	1.85 (1.19–2.85)
*Social contacts with ethnic Dutch*	
Few social contacts with ethnic Dutch	1.00
Many social contacts with ethnic Dutch	1.77 (1.15–2.71)

^a ^All associations are adjusted for age, sex, education and marital status.

### Stratification by the contextual barriers

Table 3 shows the adjusted associations of the acculturation indicators with physical activity during leisure time stratified by 1) having children, 2) occupational physical activity and 3) attractiveness of the neighbourhood. Both indicators of acculturation had a significant influence on physical activity during leisure time among the participants without children, without occupational physical activity and among participants who perceived their neighbourhood as attractive. The odds of 'Cultural orientation' were 2.17 (CI:1.22–3.85) for participants who perceived their neighbourhood as attractive, 2.18 (CI: 1.29–3.71) for the participants without children and 1.76 (CI: 1.04–2.96) for participants without physical activity at work. For 'social contacts with ethnic Dutch' we found similar associations with odds of 1.76 (CI: 1.04–2.99) for the participants who perceived their neighbourhood as attractive, 1.87 (CI: 1.11–3.14) for participants without children and 1.83 (CI: 1.08–3.10) for the participants without occupational physical activity (Table 3). In contrast, among the participants who did experience the mentioned barriers, we found no significant associations between acculturation and physical activity during leisure time. However, in some cases the odds were on a similar or even higher level than within the group without the barrier, though not significant. This was found amongst others for the effect of cultural orientation within the group with occupational physical activity.

**Table 3:** Associations^a ^of the acculturation indicators with being physically active during leisure time stratified by the contextual barriers. Presented are the Odds Ratio's (OR) of the higher acculturated groups compared to the lower acculturated groups (reference group).

	**Indicators of acculturation**	
**Contextual barriers**^c^	Cultural orientation towards the Dutch culture^b ^ OR (95% CI)	Social contacts with ethnic Dutch^b^ OR (95% CI)

**Having no children**	**2.18 (1.29–3.71)**	**1.87 (1.11–3.14)**
Children	1.28 (0.52–3.13)	1.85 (0.79–4.33)
**No occupational physical activity**	**1.76 (1.04–2.96)**	**1.83 (1.08–3.10)**
Occupational physical activity	2.21 (0.69–7.10)	0.95 (0.32–2.87)
**Attractive neighbourhood**	**2.17 (1.22–3.85)**	**1.76 (1.04–2.99)**
Less attractive neighbourhood	1.24 (0.59–2.58)	1.22 (0.55–2.73)

^a ^All associations are adjusted for age, sex, education and marital status.

^b ^The reference groups are respectively the participants with a *low *cultural orientation towards the Dutch culture and *few *social contacts with ethnic Dutch.

^c ^Figures in bold are the groups without the barrier.

**Figure 2:**
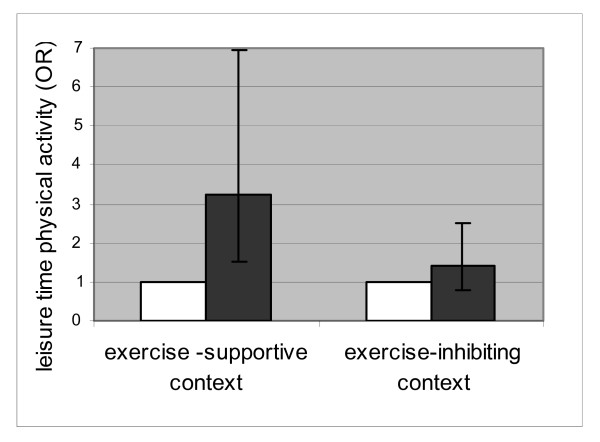
Association between 'cultural orientation towards Dutch culture' and *physical activity during leisure time *stratified by the presence of contextual barriers (no barriers = exercise-supportive context and ≥1 barrier = exercise-inhibiting context). White square: Low oriented towards Dutch culture (reference group). Black square: Highly oriented towards Dutch culture.

**Figure 3:**
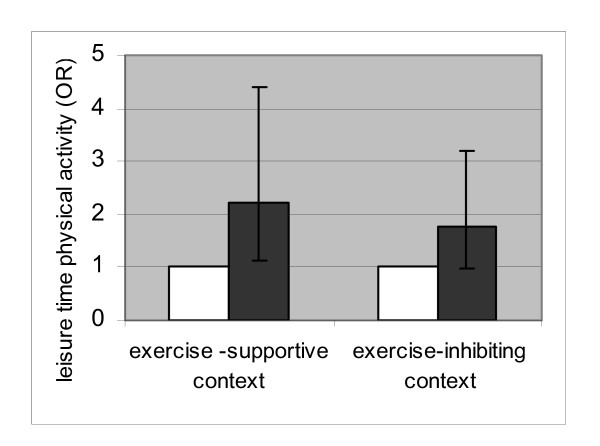
Association between 'social contacts with ethnic Dutch' and *physical activity during leisure time *stratified by the presence of contextual barriers (no barriers = exercise-supportive context and ≥1 barrier = exercise-inhibiting context). White square: Few social contacts with ethnic Dutch (reference group). Black square: Many social contacts with ethnic Dutch.

In figures 2 and 3, the associations between acculturation and physical activity during leisure time are shown for the participants in an "exercise-inhibiting context" (having at least one of the barriers) versus participants in an "exercise supportive context" (persons without any of the three barriers). This measure was calculated based on the sum score of all three barriers. Cultural orientation was significantly associated with physical activity during leisure time among the participants who did not experience any of the barriers (exercise supportive context: OR 3.24, CI: 1.51–6.96). This association was not found in the group who did experience at least one of the mentioned barriers (exercise inhibiting context: OR 1.40 (CI: 0.79–2.50). A similar, somewhat less strong, result was found for 'social contacts with ethnic Dutch' with Odds of 2.22 (CI: 1.11–4.41) within the exercise supportive context and 1.77 (CI: 0.99–3.18) within the exercise-inhibiting context.

## Discussion

This study explored how the association between acculturation and physical activity during leisure time is modified by the presence of the following contextual barriers: 'having children', 'participating in occupational physical activity' and 'living in a less attractive neighbourhood'. We found that persons with a Turkish background who were more culturally orientated towards the Dutch society and who had more social contacts with ethnic Dutch (i.e. highly acculturated), were more physically active during leisure time than persons who were less acculturated. This pattern, however, appeared to exist in particular among people without children, without participation in occupational physical activity and living in an attractive neighbourhood environment.

### Limitations

Before drawing conclusions, some limitations must be mentioned. Firstly, as in many other studies we used cross-sectional data, which implies that no causal relationship between the acculturation indicators and the outcome measure could be demonstrated. For example, the influence of social contacts with ethnic Dutch people on physical activity during leisure time could perhaps be explained by the fact that people who participate in sport have more contacts with ethnic Dutch people. In the current study however, leisure time activities also included activities that were not organized, such as cycling, walking, running in the park or doing exercises at home, which do not necessarily involve more opportunities for contact with ethnic Dutch people. In addition, the main purpose of this study was to investigate the role of the contextual factors in the association between physical activity and acculturation, therefore the cross-sectional study design will not affect our main conclusions.

Secondly, the questionnaire for assessing physical activity during leisure time, was only validated for ethnic Dutch people [42]. To minimize the chance of misinterpretation or difficulties with understanding the questions we tested the questionnaire in a pilot study among young Turkish people and we adjusted the questionnaire to include dancing as a leisure time activity as this is a popular activity among Turkish women in particular. In addition, we used trained Turkish bilingual interviewers to help in cases of difficulties answering the questions.

### Interpretations

The finding that more acculturated migrants were more physically active during their leisure time, is in line with results from other acculturation-studies among several ethnic minority populations, such as the Latino-, and Asian Americans in the US [17,18,20,51,52]. This positive association is often explained by the greater exposure to health promotion campaigns by people who speak the native language and who have many contacts with the host population. As a results their attitudes and norms towards physical activity become more similar to those in the host population. These people might also be better informed about the opportunities for physical activities, for example, how and where they can obtain access to sport facilities.

In addition to these studies, the results of the present study indicate that under certain conditions this positive effect of acculturation is not present. We illustrated that barriers such as having children (e.g. time barrier), living in al less attractive environment and participation in occupational physical activity modify the association between acculturation and physical activity during leisure time. Although several social cognitive theories that aim to explain behavioural practices, incorporated 'perceived barriers' in their model, this is often indirectly [53,54]. According to these theories, behaviour will only be performed when a person perceives no barriers that could inhibit his/her behaviour. However, none of the acculturation studies we found paid attention to the role of barriers in the association between acculturation and physical activity. This is in line with the conclusion of Hunt et al. (2004) and Salant et al. (2003), who found, in their review-study, that acculturation-studies in general, do not include contextual factors [55,56]. We suggest further study is necessary to unravel more of the potential contextual barriers that moderate the effect of acculturation on physical activity.

In contrast to most other studies we used a broader measure of acculturation than language use or language proficiency alone, instead we included items on shopping preferences, media use, and emancipation as well, in an attempt to cover a more general 'cultural orientation'. The use of 'language proficiency' or 'language use' as indicators of acculturation has been criticized by others as it would not necessarily reflect the migrants' adherence to the values and norms within the majority culture [57]. However, the majority of studies on acculturation use these indicators as they seem to be strongly related to several health related outcomes [58]. Nevertheless, we suggest that measuring different domains in which people acculturate might be a better reflection of the process of acculturation, than measuring only language use or language proficiency.

We assume that the pattern of varying influence of acculturation depending on the presence of contextual barriers, might also apply to other migrant populations. The positive association of acculturation with physical activity (during leisure time) has already been observed among different ethnic minorities and the barriers we included are acknowledged as important constraints for being physically active [17,18,20,33,34,51,52]. We further expect that our results could be generalized to older populations than those included in our study, as the barriers that we studied are in general more prevalent among adults. The majority of the older migrant adults, belong to the first generation and have on average a greater number of children and a lower socioeconomic position (e.g. more job related physical activity and living in less attractive neighbourhoods) than the young population that we studied [59].

### Implications for health promotion

Our findings provide indications to suggest that the level of physical activity during leisure time in non-Western migrant populations will not necessarily increase as a consequence of greater 'integration' in the host society among people with children, who participate in occupational physical activity or who are living in a less attractive neighbourhood. Therefore, we suggest that interventions aimed at specific ethnic groups with high prevalence rates of physical inactivity remain necessary. More specifically, our findings might imply that interventions aimed at promoting physical activity among migrants, should not only focus on the least integrated. Instead, effectiveness will probably be enhanced when interventions are sensitive to the social and environmental barriers, especially among low-income migrant populations.

## Conclusion

In conclusion, our study suggest that the influence of cultural factors, such as acculturation, might be minimized because of more dominating contextual barriers that inhibit a particular behaviour, which we illustrated with regard to physical activity during leisure time. Therefore, we recommend that in exploring the effect of the process of acculturation on physical activity, it might be necessary to take the social and physical environment into account.

## Competing interests

The author(s) declare that they have no competing interests.

## Authors' contributions

KH carried out the data-collection, performed the data-analysis and drafted the manuscript. NSK was involved in the design of the study and commented on earlier drafts of the manuscript. KS contributed to the conception and design of the study, participated in interpretation of the data and commented on drafts of the manuscripts. All authors read and approved the final manuscript.

## Pre-publication history

The pre-publication history for this paper can be accessed here:


